# Optical gain in GaAsBi/GaAs quantum well diode lasers

**DOI:** 10.1038/srep28863

**Published:** 2016-07-01

**Authors:** Igor P. Marko, Christopher A. Broderick, Shirong Jin, Peter Ludewig, Wolfgang Stolz, Kerstin Volz, Judy M. Rorison, Eoin P. O’Reilly, Stephen J. Sweeney

**Affiliations:** 1 Advanced Technology Institute and Department of Physics, University of Surrey, Guildford GU2 7XH, United Kingdom; 2 Department of Electrical and Electronic Engineering, University of Bristol, Bristol BS8 1UB, United Kingdom; 3 Materials Science Center and Faculty of Physics, Philipps-Universität Marburg, 35032 Marburg, Germany; 4 Tyndall National Institute, Lee Maltings, Dyke Parade, Cork, Ireland; 5 Department of Physics, University College Cork, Cork, Ireland

## Abstract

Electrically pumped GaAsBi/GaAs quantum well lasers are a promising new class of near-infrared devices where, by use of the unusual band structure properties of GaAsBi alloys, it is possible to suppress the dominant energy-consuming Auger recombination and inter-valence band absorption loss mechanisms, which greatly impact upon the device performance. Suppression of these loss mechanisms promises to lead to highly efficient, uncooled operation of telecommunications lasers, making GaAsBi system a strong candidate for the development of next-generation semiconductor lasers. In this report we present the first experimentally measured optical gain, absorption and spontaneous emission spectra for GaAsBi-based quantum well laser structures. We determine internal optical losses of 10–15 cm^−1^ and a peak modal gain of 24 cm^−1^, corresponding to a material gain of approximately 1500 cm^−1^ at a current density of 2 kA cm^−2^. To complement the experimental studies, a theoretical analysis of the spontaneous emission and optical gain spectra is presented, using a model based upon a 12-band **k.p** Hamiltonian for GaAsBi alloys. The results of our theoretical calculations are in excellent quantitative agreement with the experimental data, and together provide a powerful predictive capability for use in the design and optimisation of high efficiency lasers in the infrared.

Recently, there has been a concerted effort to develop high-quality GaAs-based near-infrared diode lasers using the GaAsBi material system, and in particular to push their lasing wavelengths to the datacom/telecom wavelength range of 1.3–1.6 μm. The main motivation behind this effort is due to the interesting properties of the GaAsBi alloy, which is formed by substituting relatively small quantities of bismuth in place of arsenic in GaAs^1^. Incorporation of Bi in GaAs produces a strong reduction of the band gap, *E*_g_, (by up to ~80 meV per % Bi) due to valence band anti-crossing^1^,^2^, while also strongly increasing the spin-orbit-splitting energy, Δ_SO_^3^,^4^, between the top of the valence band and the spin-orbit-split-off band^4^,^5^, something which does not occur in conventional semiconductor alloys. The unusual impact of Bi incorporation on the (In)GaAs material properties opens up a range of possibilities for practical applications in semiconductor lasers^5^, photovoltaics^6^, spintronics^3^, photodiodes^7^ and thermoelectrics^8^. Highly appealing for the development of semiconductor lasers is the possibility to grow GaAsBi laser structures such that Δ_SO_ > E_g_ in the active region. This is highly significant as it promises to suppress the dominant efficiency-limiting loss processes in near-infrared lasers, namely Auger recombination, involving the generation of “hot” holes in the spin-orbit split-off band (the so-called “CHSH” process), and inter-valence band absorption (IVBA), where emitted photons are re-absorbed in the active region, thereby increasing the internal optical losses and negatively impacting upon the laser characteristics^9^,^10^,^11^. This Δ_SO_ > E_g_ band structure is present in GaAsBi alloys containing >10% Bi, at which composition the alloy band gap is close to 1.55 μm on a GaAs substrate providing a promising route to devices such as efficient 1.55 μm monolithic vertical cavity surface emitting lasers (VCSELs)^4^,^5^. This, combined with the aforementioned ability to engineer the band structure to eliminate the dominant Auger and IVBA loss mechanisms, makes GaAsBi alloys an attractive candidate material system for the development of highly efficient, uncooled GaAs-based lasers for applications in optical communications^9^.

The first optically pumped GaAsBi based laser, consisting of a bulk-like 390 nm thick GaAs_0.975_Bi_0.025_ active layer containing 2.5% Bi grown using molecular beam epitaxy (MBE) was reported by Tominaga *et al*. and achieved optically pumped pulsed operation up to a temperature of 240 K^12^. This was followed by Ludewig *et al*. who demonstrated the first electrically pumped GaAsBi laser this time containing a quantum well (QW)-based active region^13^. This device, which was grown by metal-organic vapour phase epitaxy (MOVPE), consisted of a GaAs_0.978_Bi_0.022_ QW active region (containing 2.2% Bi), and electrically pumped pulsed operation was demonstrated at room temperature^13^. Since then the Bi composition in GaAsBi QW lasers has been increased up to 4.4% in MOVPE grown structures, although room temperature operation was not observed^14^. Using MBE growth, bulk-like GaAsBi optically pumped lasers with 5.9% Bi^15^, and electrically pumped double heterostructure lasers with up to 4% Bi, have been demonstrated^16^. To overcome the challenges associated with MBE and MOVPE growth, a hybrid approach has been developed^17^,^18^. This hybrid growth method consists of growing the QW region using MBE (thereby allowing an increased Bi composition to be achieved in the active region), while the remainder of the laser structure is grown using MOVPE (thereby maintaining the capability for rapid high quality growth of the thick waveguide and cladding layers). Using this approach, GaAsBi/GaAs lasers consisting of three QWs containing ~6% Bi have been developed, and electrically pumped operation has been demonstrated at room temperature^18^. However, characterisation of these devices has revealed performance issues related to recombination via defect states^14^. This is in line with detailed theoretical investigations of the emission dynamics^19^ and optical spectra in GaAsBi alloys, which have highlighted the strong role played by localised states (associated with Bi clustering) in determining the optical properties of bulk-like epitaxial layers. This demonstrates that despite the rapid progress that has been made in the development of this material system, there is a strong need for further improvement and optimisation of the growth and fabrication of GaAsBi materials and devices in order to realise their potential for practical applications.

A key aspect of efforts to continue the development of this material system is to develop a quantitative understanding of the impact of Bi incorporation on the properties and performance of existing devices, in order to enable the design and optimisation of longer-wavelength devices with increased Bi composition.

In addition to refinement of growth and fabrication processes, such optimisation requires a detailed understanding of the physical properties of the laser structures in order to develop improved device designs (including, e. g., specification of the QW thickness, alloy composition, strain and band offsets, as well as the number of QWs and the waveguide refractive index profile, etc.) to deliver the required laser characteristics (including high optical gain, output power and efficiency, and temperature stability).

In this report we present the first experimental measurements of the optical gain spectra of GaAsBi/(Al)GaAs QW lasers. We apply the segmented contact method^20^ to directly measure the absorption, gain and spontaneous emission (SE) spectra of a series of multi-section devices, as well as to measure the internal (cavity) optical losses in GaAsBi laser structures containing ~2% Bi. We also present a theoretical analysis of the optical properties of these devices, using a model based upon a 12-band **k.p** Hamiltonian for GaAsBi and related alloys^21^. Initial theoretical investigations of the optical gain in dilute bismide alloys^22^,^23^,^24^ have suffered from a lack of detailed information regarding the band structure of GaAsBi alloys. We have overcome these limitations by undertaking a series of detailed analyses of the electronic properties of GaAsBi alloys. On this basis we have directly parametrised the 12-band Hamiltonian of ref. 21 on the basis of (i) atomistic calculations of the alloy electronic structure, as well as (ii) detailed measurements of the electronic properties of GaAsBi QWs^25^. Based on this improved understanding of the GaAsBi band structure we have developed a theoretical model for dilute bismide QW lasers, which has been recently applied to elucidate the effects of Bi incorporation on the electronic and optical properties of ideal GaAs-based laser structures operating at wavelengths up to and including 1.55 μm^26^. Here, we apply this model to calculate the SE and optical gain spectra for a series of real GaAsBi laser structures, and compare the results of our calculations directly to experiment–the first such comparison between theory and experiment for this new class of semiconductor laser.

The experimental results we present provide the most detailed insight to date into the optical properties of QW lasers based on this novel material system. Furthermore, our calculations are in good, quantitative agreement with experiment, confirming our theoretical understanding of the unusual electronic and optical properties of GaAsBi laser structures and verifying the predictive capacity of our theoretical model for use in the design and optimisation of dilute bismide semiconductor lasers.

## Samples and Experimental Methodology

**Figure 1:**
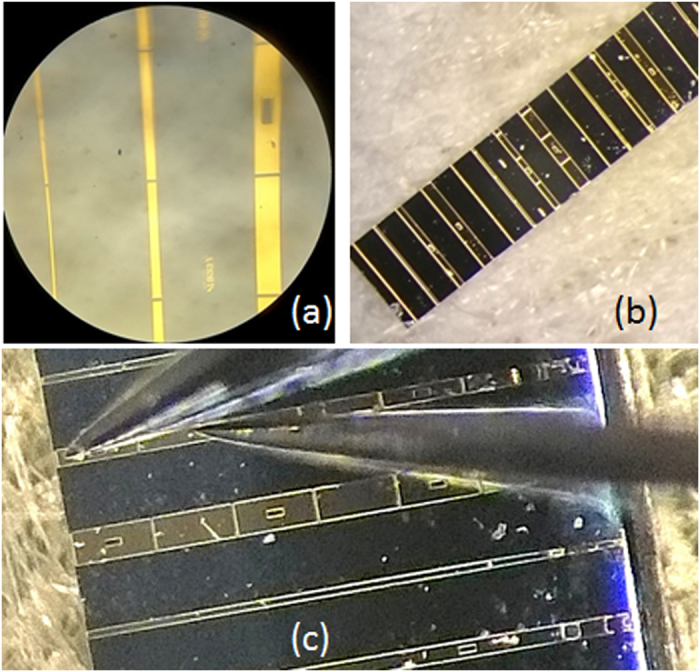
Microscope view of a series of devices used in the segmented contact measurements: (**a**) shows the segmented contact stripes, (**b**) shows a 1 mm long cleaved sample having three full contact segments (one side was cleaved at the segment end, and was then coated with a single layer of HfO_2_ anti-reflection coating), (**c**) shows a long test sample having seven segments at the edge of the wafer, with two probe tips contacting the first two segments.

A single quantum well (SQW) laser structure containing a GaAs_0.978_Bi_0.022_/AlGaAs QW was grown by MOVPE within Al_0.4_Ga_0.6_As cladding layers on an n-doped GaAs (001) substrate. A commercially available AIX 200-GFR reactor system with Pd-purified H_2_ as the carrier gas at a reduced reactor pressure of 50 mbar was used for the laser growth. The GaAsBi QW of the devices with 2.2% Bi was grown at 400 °C under pulsed precursor flow, as reported in ref. 13. Part of the material was processed into Fabry-Perot lasers, as reported elsewhere^13^,^14^,^25^. To implement the segmented contact method a special contact mask was designed to fabricate top stripe contacts divided into 300 μm long sections with ~5 μm gaps between each section, as shown in Fig. 1(a). The mask contained stripes of different width to produce 20, 50 and 100 μm wide contacts. To form the metal contacts Pt/Au were deposited on the top GaAs:Zn *p*^+^ contact layer and an AuGe/Ni/Au-based contact was deposited on the *n*^+^-substrate backside. The sample was annealed at 400 °C for 30 seconds for low-ohmic contact formation. To avoid current spreading and short-circuiting between sections, the GaAs:Zn-contact layer was etched-off using the metal stripes as a mask.

A review of different experimental techniques for optical gain measurements in diode lasers, as well as detailed methodology and advantages of the segmented contact method, is given by Blood *et al*. in ref. 20. The optical gain or modal gain, *G*, is defined as the fractional increase in energy of an optical mode per unit propagation length. It is proportional to the local material gain, *g*, with G = Γg, where Γ is the optical confinement factor defining the fractional overlap of the optical field intensity with the active QW layer(s). Denoting the total SE rate (per unit photon energy per unit area in the plane of the layer) by *I*_*spon*_, and assuming that *I*_*spon*_ is uniform along the contact stripe, the total amplified spontaneous emission (ASE) of the same polarisation emerging from a length *l* of pumped material per unit stripe width is


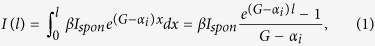


where *β* is the fraction of the SE coupled into the waveguide and *α*_*i*_ denotes the internal (cavity) optical losses^20^. Using Eq. (1), defining *C* as an extraction factor and taking into account reflection at the end facet with reflectivity *R*, the externally measured ASE spectrum can be written as


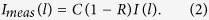


The Eq. (1) can be solved analytically for two lengths *l* = *L* and *l* = *2L*, which together with Eq. (2) gives the following expressions for the net modal gain (*G-α*_*i*_), and SE *I*_*spon*_^20^.


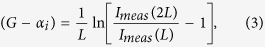



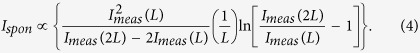


By measuring ASE spectra from the end of a single pumped section of length *L*, *I*_*meas*_(*L*), and from two pumped sections of equal length, *I*_*meas*_(*2L*), and using Eqs (3) and (4), we analysed the net modal gain and SE spectra, respectively. Furthermore, the ASE spectra measured under pumping of only the first section at the end facet, *I*_*meas*_(*1*) can be used to determine the ASE spectrum from any other sections, assuming that all sections are identical. Therefore, the ASE spectrum measured under pumping of the second section from the end facet, *I*_*meas*_(*2*), with the first segment acting as a passive absorber, will be related to *I*_*meas*_(*1*) via the modal absorption, *A* (with *A* = *Γα*, where *α* is the material absorption of the gain medium), as *I*_*meas*_(*2*) = *I*_*meas*_(*1*)exp[*−*(*A* + *α*_*i*_)*L*]^20^. From this the net modal absorption can be determined directly as


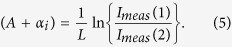


Figure 1(b) shows an example of a cleaved bar which could be used both for laser characterisation (using solid stripe contacts) and investigation of the gain and absorption spectra (using segmented contacts). To suppress round-trip amplification for a single-pass measurement the end of the segmented contact was coated with a ~115 nm thick HfO_2_ layer using a Nordiko 2000 RF magnetron sputtering system, thereby providing anti-reflection coatings, which reduce the reflectivity of the cleaved facet to R ~ 1%. During the coating deposition the top part of the sample typically gets coated as well, making it difficult to form an electrical contact to the first section. As an additional measure to stop round-trip amplification, as well as to provide single-pass measurements, we used a sample consisting of 7 sections at the edge of the wafer, as shown in Fig. 1(c). In such a configuration at least 5 segments are acting as passive absorbers and the curved wafer edge provides negligible back reflection, thereby suppressing round-trip amplification.

An optical spectrum analyser and optical power meters were used to measure the ASE from the end facet, as well as the pure SE from the QW. The latter was collected from a small 100 μm diameter window milled in the substrate (*n*) contact^14^,^27^. To avoid the effects of GaAs substrate absorption on the measured SE from the GaAsBi QW^14^,^26^, we also used a 20 μm × 50 μm window in the top (*p*) contact for the pure SE measurements. At low currents (up to 50 mA, or J = 330 A cm^−2^) electrical pumping was provided using a continuous wave (CW) source-meter unit, while at higher currents a pulsed voltage source was used to provide 500 ns long pulses at a frequency of 20 kHz, in order to avoid self-heating effects and to maximise the signal-to-noise ratio.

## Theoretical Modelling

Due to the large differences in size and chemical properties between Bi atoms and the As atoms they replace, Bi acts as an isovalent impurity when incorporated into GaAs in dilute concentrations to form the GaAsBi alloy. It is this impurity-like behaviour of Bi atoms in GaAs and related III-V semiconductor matrices that gives rise to the unusual electronic properties of dilute bismide alloys, and significantly complicates the theoretical description of the material band structure^2^,^28^.

We have previously developed an atomistic tight-binding model for GaAsBi alloys, which we have applied successfully to develop a quantitative understanding of the electronic, optical and spin properties of this unusual class of semiconductor materials^29^,^30^,^31^,^32^. Based on these analyses we demonstrated that substitutional Bi atoms form highly localised resonant impurity states in the GaAs valence band, as well as a range of bound states corresponding to pairs and larger clusters formed by Bi atoms located at second nearest-neighbour anion lattice sites relative to one another. Despite the extremely complicated valence band structure of a realistic, disordered GaAsBi alloy, further analyses we have undertaken have verified explicitly that the general features of the GaAsBi alloy band structure are determined in large part by the effects of the resonant states associated with isolated substitutional Bi atoms. In ref. 21 we showed in detail how these general electronic properties can be understood quantitatively in terms of a composition-dependent band-anticrossing interaction between these localised resonant impurity states, and the extended valence band edge states of the host matrix semiconductor.

Based on these insights, we have used a series of detailed supercell calculations to derive an extended 12-band **k.p** Hamiltonian for GaAsBi alloys^21^,^33^. The extended basis set of this Hamiltonian contains the spin-degenerate, zone-centre Bloch states of the GaAs host matrix conduction, heavy-hole, light-hole and spin-split-off-hole bands, in addition to the aforementioned Bi-related resonant impurity states. In ref. 25 we applied the 12-band model to the study of the band structure of GaAsBi QWs and, by comparison with the results of polarisation-resolved photo-voltage measurements, were able to extract information about and constrain the structural and band structure parameters of the QW and barrier materials, as well as the QW band offsets.

Combining the 12-band **k.p** Hamiltonian of ref. 21 with the constrained material parameters and band offsets of ref. 25 we have developed a theoretical model of the electronic and optical properties of dilute bismide QW lasers^26^. Our theoretical model is based on a numerically efficient reciprocal space (plane wave) approach to the calculation of the QW band structure, and includes electrostatic effects via self-consistent computation of the electronic structure with coupling to Poisson’s equation for the charge density generated by the electron and hole populations (which are assumed to be independently in quasi-equilibrium). The model also explicitly incorporates key band structure effects in the computation of the electronic and optical properties, including: Bi-induced hybridisation and carrier localisation, temperature- and injection-dependent carrier spillout from the QW(s), band mixing and pseudomorphic strain. Full details of our theoretical model for GaAsBi lasers, as well as a detailed analysis of the impact of Bi incorporation on the material and device properties of GaAs-based dilute bismide semiconductor lasers, can be found in ref. 26.

We apply this theoretical model below to compute, analyse and compare to experiment, the SE and optical gain spectra of the GaAsBi SQW laser structures described in *the previous section*. To facilitate the analysis to be presented in *section* “*Results and Discussion*”, we briefly discuss here the calculation of the material gain. Our calculation of the material gain spectrum for a given polarisation follows the approach of ref. 34, which consists of transforming the corresponding polarisation component of the SE rate according to


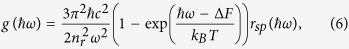


where Δ*F* is the quasi-Fermi level separation at temperature *T* in the presence of an injected carrier density *n*, *n*_r_ is the refractive index of the active region, and *r*_sp_ is the SE rate (calculated as described in ref. 26). In this work we are concerned solely with TE-polarised gain spectra, and so *r*_sp_ is understood to represent only the TE-polarised component of the total SE rate (which gives rise to the leading factor of 3/2 in Eq. (6)). This approach to calculating the material gain spectrum has the benefits that (i) it removes the anomalous absorption at energies below the active region band gap that can plague calculations undertaken using an energy- and crystal momentum-independent interband relaxation time (homogeneous spectral linewidth), and (ii) by definition, *g*(Δ*F*) = 0, so that the transparency point on the high energy side of the gain peak is maintained at the correct, thermodynamically-consistent energy. As we will see below, this latter characteristic of Eq. (6) will enable us to associate a distinct carrier density *n* in the theoretical calculations with each current density *J* at which the gain spectrum was measured, and hence to compare the results of our theoretical calculations directly to the experimental gain spectra.

## Results and Discussion

**Figure 2:**
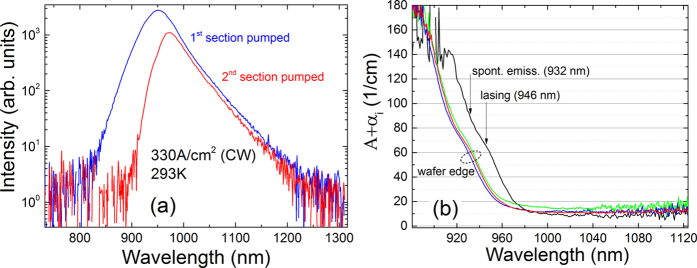
(**a**) Amplified spontaneous emission spectra measured under pumping of the first section from the device end, *I*_*meas*_(*1*) (solid blue line), and under pumping of the second section from the device end, *I*_*meas*_(*2*) (with the first section acting as a passive absorber; solid red line). (**b**) Net modal absorption spectra obtained using Eq. (5) for a series of different devices: a device fabricated from material located 5–10 mm from the wafer edge (cf. Fig. 1(b); solid black line), and devices fabricated from material located ~2 mm from the wafer edge (cf. Fig. 1(c); red, blue and green solid lines, respectively).

We earlier reported on the characterisation and performance of Fabry-Perot lasers fabricated from these GaAs_0.978_Bi_0.022_/AlGaAs SQW laser structures^13^,^14^,^25^. We begin here by considering the modal absorption spectra, which were obtained by measuring ASE spectra (cf. Eq. (2)) under pumping only the first, *I*_*meas*_(*1*) (equivalent to *I*_*meas*_(*L*) here), and then the second section, *I*_*meas*_(*2*), from the sample facet (cf. Eq.(5)). These measurements, shown in Fig. 2(a), were carried out using a relatively low continuous current of 50 mA (corresponding to a current density J = 330 A cm^−2^), thereby avoiding self-heating effects and providing a low-noise signal compared to pulsed measurements. The modal absorption spectra measured in this manner are shown in Fig. 2(b), for a device fabricated from material located ~5–10 mm away from the wafer edge and for devices fabricated from material located ~2 mm away from the wafer edge.

Previous polarisation-resolved photo-voltage measurements we have undertaken on a series of GaAsBi laser structures having nominally identical Bi compositions of 2.2% revealed the presence of Bi compositional variations across a given wafer, where a variation of approximately 30 meV was observed in the QW band gap (corresponding in theoretical calculations to Bi compositional variations of up to ±0.4%)^25^. The measured absorption spectra presented in Fig. 2(b) are consistent with these measurements: the absorption edge measured for the device fabricated from material located ~5–10 mm from the wafer edge is red-shifted by approximately 20 nm compared to the absorption edges measured for the devices fabricated from material located ~2 mm away from the wafer edge. As we will see in our discussion of the measured SE spectra for these devices below, this variation of the absorption edge across the wafer corresponds well to our previously calculated Bi compositional variations for laser structures containing ~2% Bi^25^. Furthermore, we note that the absorption edge depicted by the black line in Fig. 2(b) is in good agreement with the previously measured SE peak and lasing wavelengths for Fabry-Perot lasers fabricated from the same GaAs_0.978_Bi_0.022_ QW material (932 and 946 nm respectively^13^,^14^, denoted by arrows in Fig. 2(b)).

The measured modal absorption of approximately 100 cm^−1^ close to 930 nm corresponds to a material absorption coefficient at this wavelength of 6025 cm^−1^, based on an optical confinement factor Γ = 1.66% calculated for a GaAs_0.978_Bi_0.022_ SQW laser structure^26^. The constant value of the net modal absorption (*A* + *α*_*i*_) at longer wavelengths below the QW band gap (>1000 nm, with *A* = 0) enables us to extract the optical (cavity) losses. Based on the data shown in Fig. 2(b) we find *α*_*i*_ = 10–15 cm^−1^ in the devices studied, with the larger values of *α*_*i*_corresponding to devices fabricated from material located closer to the wafer edge. We note that these optical losses are relatively small, indicating generally good optical quality of the fabricated waveguides.

**Figure 3:**
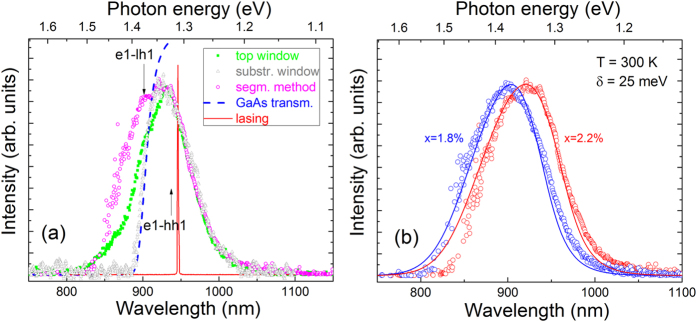
(**a**) SE spectra at the threshold current density (*J*_th_ ≈ 1.6 kAcm^−2^), measured from a window milled in the substrate contact (open grey circles), a window milled in the top contact (closed green squares), and using the segmented contact method (cf. Eq. (4); open pink circles). The dashed blue line depicts the GaAs substrate transmission spectrum. (**b**) Measured (using the segmented contact method; open circles) and calculated (solid lines) SE spectra at threshold for a device fabricated from material located ~2 mm from the wafer edge (blue) and fabricated from material located 5–10 mm from the wafer edge (red).

Next, we turn our attention to the measured SE spectra from these devices, which can also be obtained using the segmented contact method (cf. Eq. (4)), and compare the SE spectra measured using this approach to our previous measurements in which the SE was collected from a window milled in the substrate contact^14^,^26^. The advantage of using the segmented contact method to measure the SE from the end facet is that both the TE (in-plane) and TM (out-of-plane) polarised components of the SE can be collected, whereas TM-polarised light, which propagates along the laser cavity only, cannot be collected through a window milled in either the top (*p*) or substrate (*n*) contacts. Because of this, the integrated SE collected through a contact window can underestimate the radiative current density (*J*_rad_) in the device if there is appreciable TM-polarised emission. For comparative purposes, SE spectra were also measured from a window milled in the top contact in order to remove the effect of substrate (GaAs) absorption, which has been previously shown to encroach on the shorter wavelength (higher energy) SE^14^,^26^. Figure 3(a) shows the SE spectra measured using these three approaches, for devices fabricated from material located ~5–10 mm away from the wafer edge, all of which we found to have the SE peak at the same wavelength. The measurements were performed at the threshold current density *J*_th_ ~ 1.6 kAcm^−2 ^14.

The open grey triangles, closed green squares and open pink circles in Fig. 3(a) show the SE spectra measured respectively from the top and substrate windows, and using the segmented contact method. Also shown in Fig. 3(a) are a typical lasing spectrum (solid red line) and the measured substrate (GaAs) band edge transmission (dashed blue line). Examining the SE spectra measured using these three approaches we note that the SE measured from the substrate window is most affected by absorption in the (relatively thick) GaAs substrate, as highlighted by comparison with the GaAs band edge transmission. The SE measured from the top contact window does not undergo significant absorption, and hence is broader at shorter wavelengths and has a more symmetrical overall shape. The peak wavelengths of these two spectra are virtually identical, and are in good agreement with the calculated QW band gap of 1.323 eV (937 nm)–i.e. the transition energy between the lowest energy electron and highest energy hole states in the QW (*e*1-*hh*1, since the highest energy hole state is calculated to be heavy-hole-like at the QW Brillouin zone centre). Interestingly the SE spectrum measured using the segmented contact method, while virtually identical to the contact window measurements at the band edge (longer wavelengths), is significantly broader at shorter wavelengths. In particular, we observe a second peak in the SE spectrum obtained using Eq. (4) at 905 nm, which is in good agreement with the calculated transition energy between *e*1 and the second-highest energy hole state (*lh*1, which is calculated to be predominantly light-hole-like at the QW Brillouin zone centre) 1.375 eV (902 nm). Based on this, as well as our calculations of the full SE spectra (to be discussed below), we therefore conclude that the excess high energy SE observed in the segmented contact measurements is attributable to TM-polarised radiative recombination of electrons occupying *e*1 conduction states with holes occupying *lh*1 valence states, which, as described above, is not possible to detect in measurements taken from a contact window.

Comparing the integrated SE for the spectra shown in Fig. 3(a) we find that they are in the ratio 100:83:71, for measurements using the segmented contact method, as well as from the top and substrate contact windows, respectively. We note that this discrepancy in the ratios of the integrated SE, and the associated underestimation of *J*_rad_ from the window measurements, will tend to increase with increasing temperature due to broadening of the SE spectra as the distribution in energy of the carriers in the conduction and valence bands increases (leading to a larger portion of the high energy SE measured from the substrate contact window being absorbed by the GaAs substrate before it can be collected in the experiment). In ref. 14 we used a linear interpolation of low temperature data, where the contribution to *J*_rad_ from transitions involving *lh1* states as well as the effect of GaAs substrate absorption are negligible, to estimate *J*_rad_ at room temperature, which gave an approximately 45% greater value than that obtained using the integrated SE from the substrate window. Using the segmented contact approach, we confirm that the value of *J*_*rad*_ determined using the integrated SE measured from the substrate window is indeed underestimated by approximately 30% at room temperature due to the combined effects of GaAs substrate absorption and the undetected TM-polarised emission due to carrier recombination involving light-hole-like states. This is an important consideration for future SE measurements.

Figure 3(b) shows the SE spectra measured at threshold using the segmented contact method for devices fabricated from material located away from the wafer edge (open red circles) and close to the wafer edge (open blue circles). Both spectra have similar overall shape with the main difference between them being the wavelength of the emission peak, which is approximately 20 nm shorter in the device fabricated from material located close to the wafer edge. We note that this shifted emission peak is consistent with the shift in the absorption edge across the wafer (cf. Fig. 2(b)), as well as with the experimental data presented in ref. 25, and indicates that the Bi composition is slightly lower in the QW material at the wafer edge than closer to the centre of the wafer. In order to quantify this variation in Bi composition across the wafer, and to facilitate our theoretical analysis of the optical gain below, we have used the theoretical model described in *section* “*Theoretical Modelling*” to analyse the SE spectra shown in Fig. 3(b). Through our theoretical calculations we find that the measured SE peak wavelength of 910 nm at the wafer edge corresponds to a Bi composition of 1.8% in the QW (assuming in the theoretical calculations that the QW width is fixed at its nominal value of 6.4 nm^25^).

Next, by comparing the full calculated SE spectrum to the experimental data, we find that the spectral broadening is best described by a hyperbolic secant lineshape of the form 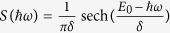, with a linewidth δ = 25 meV. We note that this lineshape function was previously found to describe well the SE spectra of 1.3 μm GaInNAs dilute nitride QW lasers, and that the best-fit linewidth of 17 meV at room temperature for the device of ref. 35 is similar to that obtained here. Using this lineshape and linewidth, the calculated SE spectrum for the wafer-edge device is shown in Fig. 3(b) using a solid blue line. The theoretical calculation was also carried out at the threshold injection level, which was determined as the carrier density required to produce the TE-polarised threshold material gain of 1325 cm^−1^ (obtained using an effective index calculation to determine an optical confinement factor of 1.60% for a QW containing 1.8% Bi, and using the measured threshold current density of 1.6 kA cm^−2^ in similar devices^14^). Due to uncertainty in the absolute units and relative intensity of the SE measurements performed on different devices, we have normalised the calculated SE spectrum to its measured value at the SE peak in order to compare the theoretical and experimental data. We note that the measured and calculated SE spectra are in good overall agreement, with the spectral broadening observed in the experiment well described by a combination of the energy broadening of the electron and hole (quasi-Fermi) distribution functions at room temperature, as well as the aforementioned hyperbolic secant line broadening.

In order to quantify the Bi compositional variation across the wafer we have also calculated the SE spectrum for the device fabricated from material located away from the edge of the wafer. The calculation of the SE spectrum in this case proceeds as above, this time with the Bi composition being the only parameter allowed to vary. The result of this calculation is shown using a solid red line in Fig. 3(b). We find that the measured SE peak at 932 nm is well described in the theoretical calculations assuming that the QW contains the nominal Bi composition of 2.2%, and that the calculated SE spectrum at this composition is generally in good overall agreement with experiment for this device. Overall, these theoretical results suggest that the Bi composition in the QW is close to the nominal value of 2.2% across the central part of the wafer, but it is slightly reduced to 1.8% towards the wafer edges. The reduction of the Bi composition towards the wafer edge is possible if the temperature during growth is lower at the edges compared to the centre of the wafer; however, since the gas phase is optimized for 400 °C with relatively high V/III ratio a reduction of the Bi composition cannot be excluded. We recall that this variation in Bi composition is consistent with that determined previously via photo-voltage measurements performed on a series of MOVPE-grown laser structures having the same nominal Bi composition of 2.2%^25^.

**Figure 4:**
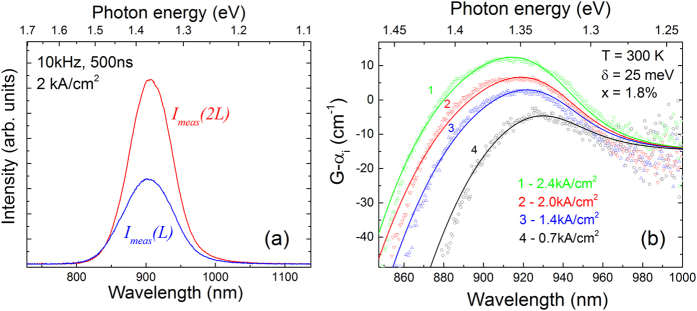
(**a**) Measured ASE spectra from the end facet of the device shown in Fig. 1(c), under pumping of a single section, *I*_meas_(*L*) (solid blue line), and under pumping of two adjacent sections of the same length, *I*_meas_(2*L*) (solid red line). (**b**) Measured (using the segmented contact method, cf. Eq. (3); open symbols) and calculated (cf. Eq. (6); solid lines) net modal gain spectra for the same device, at injected current densities of *J* = 0.7, 1.4, 2.0 and 2.4 kA/cm^2^ (black, blue, red and green circles/lines respectively). The corresponding carrier densities in the theoretical calculations, determined as outlined in the text, are *n* = 5.12, 7.11, 8.24 and 9.38 × 10^18^ cm^−3^.

Figure 4(a) shows exemplar ASE spectra measured from the wafer-edge device facet under pumping of a single section *I*_meas_(*L*) (solid blue line), and under pumping of two sections *I*_meas_(2*L*) (solid red line), at the threshold current density. By performing such measurements at different current densities and using Eq. (3) we obtain the net modal gain spectra shown in Fig. 4(b), which were measured at current densities of 0.7, 1.4, 2.0 and 2.4 kAcm^−2^ (shown using black, blue, red and green open symbols, respectively). The measured gain spectra are relatively broad, with a full width at half maximum of approximately 100 meV at a current density of 2 kAcm^−2^, which is close to twice that observed for an InGaAs/GaAs SQW laser operating at a similar wavelength^36^ and is most likely related to the strong Bi-induced inhomogeneous broadening that is characteristic of the optical spectra of GaAsBi alloys^30^. Based on the α_i_ = 15 cm^−1^ optical losses measured for the device fabricated from material located close to the wafer edge (cf. Fig. 2(b)) the estimated peak modal gain at *J* = 2 kAcm^−2^ is *G*_peak_ = 24 cm^−1^. Using the calculated optical confinement factor of 1.60% for this GaAs_0.982_Bi_0.018_ SQW laser structure we estimate a peak material gain *g*_peak_ ≈ 1500 cm^−1^ at *J* = 2 kA cm^−2^, which agrees well with the calculated value of 1560 cm^−1^.

In order to compare our theoretical gain spectra to experiment we must determine the carrier density corresponding to each current density at which the gain spectra depicted by open symbols in Fig. 4(b) were measured. In order to do this we recall from Eq. (6) that the transparency point at which there is zero material/modal gain on the high energy side of the gain peak is given when the photon energy is equal to the quasi-Fermi level separation at a given level of injection. We therefore proceed by shifting the measured net modal gain spectra upwards by the α_i_ = 15 cm^−1^ optical losses in order to obtain the absolute modal gain *G* in the device. Next, we use the transparency points on each of these absolute modal gain spectra to extract the quasi-Fermi level separation Δ*F* corresponding to each current density in the experiment. Finally, using our theoretical model we calculate Δ*F* as a function of the injected carrier density *n* and, in doing so, determine the value of *n* which produces the extracted Δ*F* for each current density *J*. Following this procedure, we find that the current densities of 0.7, 1.4, 2.0 and 2.4 kA cm^−2^, at which the gain spectra were measured, correspond to quasi-Fermi level separations of 1.375, 1.409, 1.423 and 1.435 eV, which in turn correspond respectively to carrier densities in the theoretical calculations of 5.12, 7.11, 8.24 and 9.38 × 10^18^ cm^−3^. We then calculated the TE-polarised component of the SE rate at each of these carrier densities as outlined above, from which the theoretical net modal gain spectrum was determined at each carrier density as *Γg* − *α*_*i*_, with Γ = 1.60% and α_i_ = 15 cm^−1^, and where *g* is the TE-polarised material gain spectrum obtained from the SE rate using Eq. (6). The results of these calculations are shown in Fig. 4(b) using, in order of increasing current/carrier density, black, blue, red and green solid lines. We note that the theoretical gain spectra shown using black, blue and red solid lines include *e*1-*hh*1 optical transitions only while the green solid line, corresponding to the highest current density of 2.4 kA cm^−2^, also includes *e*1-*lh*1 optical transitions. Including *e1-hh1* transitions only at the highest carrier density was found to underestimate the peak modal gain by ~10%, highlighting that TE-polarised optical recombination involving light-hole-like states becomes important at higher levels of injection. Overall, we see that the theoretical spectra are in good, quantitative agreement with experiment: the calculated magnitude of the net modal gain is in excellent agreement with that measured using the segmented contact method across the full investigated range of current densities (confirming the accuracy of the optical transition matrix elements derived within the framework of the 12-band **k.p** model^26^), and the overall shape of the experimental gain spectrum is well reproduced at each current/carrier density by our theoretical model.

**Figure 5:**
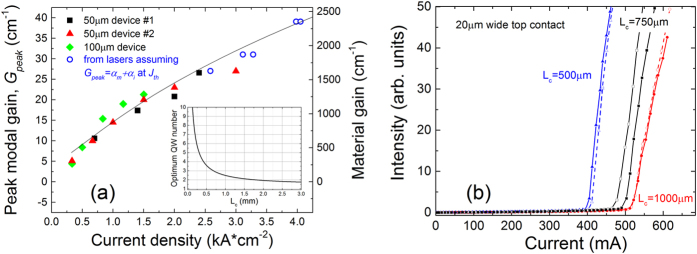
(**a**) Measured (various symbols) and calculated (solid black line) variation of the peak/threshold modal gain as a function of injected current density for a range of multi-section (closed symbols) and Fabry-Perot (open symbols) devices. The peak gain data for the multi-section devices were determined from segmented contact measurements of the gain spectra. Closed black squares and red triangles (green diamonds) show the data measured for multi-section devices having 50 (100) μm wide contact stripes. The threshold gain data for the Fabry-Perot devices (open blue circles) were determined as the sum of the cavity and mirror losses at threshold from the measured light output vs. current (L-I) characteristics for the devices. The inset in (**a**) shows the estimated variation of the number of QWs required to minimise the *J*_*th*_ for this series of devices, as a function of cavity length. (**b**) Measured L-I characteristics for the series of Fabry-Perot devices, from which the threshold gain data in (**a**) were obtained.

From measurements of the gain spectra for several devices, fabricated from material located at the wafer edge, we present in Fig. 5(a) the variation of the peak modal gain *G*_peak_ (closed symbols) as well as the threshold modal gain *G*_*th*_ (open symbols) as a function of current density, where the latter was determined as the sum of the cavity (*α*_*i*_) and mirror (*α*_*m*_) losses, *G*_th_ = *α*_*i*_ + *α*_*m*_, which is equal to the peak modal gain at threshold determined from the light output vs. current (L-I) characteristics for a series of Fabry-Perot devices with 20 μm wide contacts and cavity lengths *L*_c_ = 0.5–1 mm (shown in Fig. 5(b)). As these Fabry-Perot devices were fabricated from the same part of the wafer as the devices for which the SE and gain spectra of Fig. 4(a,b) were measured, it was assumed that they have the same *α*_*i*_ = 15 cm^−1^ optical losses; the facet (mirror) losses were calculated in each case as *α*_*m*_ = (*1/L*_*c*_)ln(*1/R*), where *R* is the facet reflectivity. Returning to Fig. 5(a), we note that the peak modal gain data obtained in this manner for the Fabry-Perot devices (open symbols) fit well with the overall trend observed for the multi-section devices upon which the segmented contact measurements were performed (closed symbols).

Based on the correspondence between the experimental current and theoretical carrier densities determined in our analysis of the gain measurements shown in Fig. 4(b), we have used our theoretical model to calculate the variation of *G*_peak_ with *J*. The results of this calculation are depicted by the solid black line in Fig. 5(a). We see that the theoretical calculation is again in good, quantitative agreement with the experimental data, confirming that the theoretical model is capable of describing the GaAsBi gain spectra across a wide range of current densities, as well as for a variety of multi-section and Fabry-Perot devices. The inset in Fig. 5(a) presents a theoretical estimate of the optimum number of QWs, *N*_opt_, required to minimise the threshold current density *J*_th_ as a function of *L*_c_. This optimum number of QWs was calculated as *N*_opt_ = (α_i_ + α_m_)/*G*_0,_ where the modal gain *G*_0_ = 10.9 cm^−1^ corresponds to the maximum ratio *G*/*J* required to minimise *J*_th_^37^. *G*_*0*_ was determined as the point at which a line through *G* = 0 is tangent to a fit to the measured *G*_peak_ vs. *J* data^37^. Note that while this provides a reasonable estimate for low numbers of QWs it is generally less accurate for a higher number of QWs, due to non-uniform pumping and poorer hole transport. Notwithstanding this, the calculated variation of *N*_opt_ with *L*_c_ corresponds well to the results presented in ref. 14, in which we observed a reduction by close to a factor of two in the threshold current density per QW in going from an SQW device to one containing three QWs (for a fixed cavity length *L*_c_ = 1 mm).

## Conclusion

In this report we have presented the first experimental measurements of optical gain in dilute bismide semiconductor laser structures. We used the segmented contact method to study the optical properties of a series of GaAsBi SQW laser structures containing ~2% Bi. Using this approach we were able to directly measure the absorption, SE, and optical gain spectra, as well as to determine the optical (cavity) losses in the laser structures (the values of which we found to be in the range of 10–15 cm^−1^). In addition to confirming the well-known Bi-induced red-shift of the optical absorption and emission peak wavelengths, our experimental measurements also indicate that transitions involving higher energy light-hole-like valence states make appreciable contributions to the SE and optical gain at higher injection levels (*J* > 2 kA cm^−2^).

In conjunction with these experimental measurements, we have also used theoretical calculations to facilitate our analysis of the laser devices. Beginning from a 12-band **k.p** model which has previously been constrained against detailed measurements of the electronic properties of GaAsBi QW laser structures, we analysed the SE from several devices. This enabled us to identify and quantify (i) Bi composition variations across the wafer from which the laser devices were fabricated, and (ii) Bi-induced inhomogeneous broadening of the optical spectra. Our calculations of the SE and optical gain spectra, as well as the peak modal gain as a function of current density, were found to be in good, quantitative agreement with experiment across a wide range of current densities for a range of multi-section and Fabry-Perot devices. This approach provides a powerful predictive capability for the design and optimisation of future bismide-based devices, including in particular highly efficient, cooler-free GaAs-based long-wavelength lasers.

## Additional Information

**How to cite this article**: Marko, I. P. *et al*. Optical gain in GaAsBi/GaAs quantum well diode lasers. *Sci. Rep*. **6**, 28863; doi: 10.1038/srep28863 (2016).
